# Self-Reported Workplace Injuries Among Informal Waste Pickers in Landfill Sites in Johannesburg, South Africa

**DOI:** 10.3390/ijerph23040509

**Published:** 2026-04-16

**Authors:** Hlologelo Ramatsoma, Jeanneth Manganyi, Keneilwe Ditema, Nisha Naicker

**Affiliations:** 1 National Institute for Occupational Health, National Health Laboratory Service, Johannesburg 2001, South Africa; hlologelor@nioh.ac.za (H.R.); jeannethm@nioh.ac.za (J.M.); 2 School of Public Health, Faculty of Health Sciences, University of the Witwatersrand, Johannesburg 2193, South Africa; 3 Department of Environmental Health, University of Johannesburg, Johannesburg 2028, South Africa; kmditema@gmail.com

**Keywords:** waste pickers, landfill sites, injuries, workplace injuries, occupational injuries

## Abstract

**Highlights:**

**Public health relevance—How does this work relate to a public health issue?**

- Informal waste picking is a vital yet hazardous component of the circular economy, where a vulnerable, marginalised workforce faces occupational risks within unregulated landfill environments.
- This study documents the high prevalence of work-related injuries, including lacerations and needle-stick injuries, among landfill-based waste pickers, consistent with exposures to sharp materials and mixed waste streams in the absence of adequate protective measures.

**Public health significance—Why is this work of significance to public health?**

- This research provides evidence that landfill-based waste pickers experience a higher injury prevalence (86.2%), exceeding that reported in semi-formalised recycling settings; however, differences in study population, injury definition, and data collection period preclude direct causal attribution to the landfill environment alone. The findings suggest a convergence of mechanical, environmental, and social injury types in this setting.

**Public health implications—What are the key implications or messages for practitioners, policy makers and/or researchers in public health?**

- Municipal authorities should move beyond basic safety awareness by formally integrating informal pickers into occupational health and safety management frameworks and providing mandatory, task-appropriate, puncture-resistant PPE through site management structures rather than relying on workers to source their own equipment.
- Practitioners and researchers should consider interventions that address occupational and structural risk factors, including interpersonal violence, biological hazards, and inadequate PPE provision, to protect this essential but currently unregulated workforce better.

**Abstract:**

While South Africa’s recycling chain relies heavily on informal labour, the burden of non-fatal workplace injuries among landfill-based waste pickers remains poorly characterised. This study aimed to estimate the prevalence of self-reported non-fatal workplace injuries and identify associated factors among informal waste pickers at landfill sites in Johannesburg, South Africa. We conducted a cross-sectional study at two purposively selected landfill sites in Johannesburg. Using convenience sampling, 354 waste pickers were enrolled (median age 34 years; 73.2% male). A structured questionnaire captured worker characteristics and self-reported injuries over the preceding six months. Robust (modified) Poisson regression was utilised to determine associations with self-reported workplace injury. Overall, 86.2% of participants reported at least one injury. Lacerations caused by contact with waste materials predominated (82.7%), followed by violence (20.5%) and needle-stick injuries (19.9%). Notably, 94.1% of participants reported using personal protective equipment (PPE), yet the injury prevalence was high. In the multivariable model, each additional year of landfill work experience was associated with a 1.0% higher prevalence of reported injury (adjusted prevalence ratio [aPR] 1.01; 95% CI 1.01–1.02). Conversely, pickers aged 51 years and older had a 32% lower prevalence of injury than those aged 18–28 (aPR 0.68; 95% CI 0.51–0.90). To mitigate these risks, municipal authorities should implement mandatory safety training for site entry, provide industrial-grade, puncture-resistant PPE, and formalise the integration of landfill pickers into institutional occupational health frameworks.

## 1. Introduction

South Africa’s rapid urban growth and persistent unemployment have fostered a large informal waste-recovery sector. Johannesburg generates an estimated 1.4 to 1.6 million tonnes of municipal solid waste annually, the majority of which is disposed of in landfill sites [1,2]. Against a backdrop of unemployment estimated at 27.1% at the time of this study [3], approximately 90,000 individuals nationwide rely on waste picking as a primary source of income; however, the true figure is likely higher [4].

Despite their economic marginalisation; waste pickers are responsible for diverting over 90% of recyclables that enter the formal recycling stream [4], and their labour directly reduces landfill volumes and associated greenhouse gas emissions. The profession of waste picking at landfill sites is attractive to many due to minimal entry barriers and the consistent market value of recovered materials; however, this accessibility comes at a cost. Waste pickers are a highly vulnerable demographic, operating within an informal industry that offers no legal or social protections while enduring pervasive social stigma and discrimination [5].

The day-to-day duties of landfill waste pickers involve multiple injury-relevant exposures. Pickers routinely spot incoming waste trucks to gain early access to high-value materials, placing them in direct proximity to heavy vehicles and thereby elevating the risk of vehicle-related injuries. Once waste is disposed, intensive manual sorting through mixed, unsegregated waste increases exposure to sharp materials, broken glass, and discarded sharp medical waste. On-site processing and manual hauling of bulk bags across uneven terrain increase musculoskeletal strain and fall-related risk [5,6].

As landfill capacity nears exhaustion, the intensity of picking has increased. Yet the specific dynamics of these large-scale sites remain under-researched compared to semi-formal hubs like Buy-Back Centres (BBCs), where injury prevalence was recently recorded at 69.4% [7]. Evidence from other developing nations suggests that the prevalence of injuries on landfill sites is much higher; for example, a study in Nigeria found that 78.6% of landfill pickers reported work-related injuries, with 37.8% of those cases involving cuts from sharp objects such as razor blades [8].

It is important to understand the injury profile of these landfill-based waste pickers within the broader socio-demographic context in which they operate. Many reside in informal settlements adjacent to or directly on landfill sites, where conditions, including limited access to clean water, inadequate sanitation, and persistent food insecurity, may compound health vulnerability [9]. While the pathways from these structural conditions to occupational injury were not directly measured in the present study, residential proximity to hazardous waste environments may exacerbate the physical risks of landfill work and reduce recovery capacity following injury.

Despite the physically hazardous nature of landfill-based waste picking, the occupational injury burden among this specific workforce in South Africa remains poorly characterised. Previous research has predominantly focused on general health outcomes [10] or on workers in semi-formalised settings such as buy-back centres, which differ substantially from landfill environments in waste composition, machinery exposure, and regulatory oversight [7]. This study, therefore, aimed to estimate the prevalence of non-fatal self-reported occupational injuries and to identify socio-demographic and occupational factors associated with injury among informal waste pickers at landfill sites in Johannesburg, South Africa.

## 2. Materials and Methods

### 2.1. Study Design and Setting

A cross-sectional study was conducted between March and April 2018 at two of the four operational municipal landfill sites in Johannesburg, Gauteng, South Africa. The two largest sites were purposively selected based on their high waste-picker density and their location within the city’s most populous areas. The south-western facility hosts an estimated 3000 informal pickers; the western facility is smaller, hosting approximately 600 waste pickers.

Prior to data collection, a qualitative health risk assessment of both sites was conducted by a trained occupational hygienist to qualify the occupational exposure. The assessment followed the ‘Five Steps to Risk Assessment’ tool developed by the UK Health and Safety Executive (HSE) [11]. Key hazards identified and evaluated at both sites included: (1) airborne dust generated by incoming dump trucks and compactors, carrying organic matter with potential for skin irritation, respiratory irritation, allergic reactions, and pathogen exposure, with the majority of workers observed not wearing dust masks, instead using garments or scarves as improvised respiratory protection; and (2) hazardous chemical substances present in general household waste, including organic dust, pesticides, and organic solvents, with no adequate inhalation control measures observed and workers similarly reliant on improvised face coverings [12].

More granular site-level operational data, including precise waste intake volumes, formal machinery inventories, and management structures, were unavailable, reflecting the undocumented and largely unregulated nature of informal landfill work in this setting.

### 2.2. Study Population and Sample

The study population comprised informal waste pickers of both genders, aged 18 years and older, who were actively working at these locations. A target sample of 365 participants was calculated using a single-proportion formula (n = Z^2^ × p(1 − p)/d^2^), based on a 95% confidence level, a 5% margin of error, and an expected prevalence of laceration injuries of 39% derived from a comparable developing-country context [8]. This laceration-specific estimate was used as an approximation for the broader outcome of any self-reported workplace injury, as no prior local estimate was available. A stratified proportional allocation strategy was employed to ensure that the sample reflected the population distribution across the two sites. Population size estimates were obtained from the landfill site managers. Based on these figures, the south-western facility, which hosts an estimated 3000 waste pickers, accounted for 82% of the total sample (n = 299) and the western facility, hosting approximately 600 waste pickers, accounted for the remaining 18% (n = 66). Within each site, participants were recruited through non-probabilistic convenience sampling. Of the 365 participants, 354 were included in this analysis, with 11 removed due to missing outcome information.

### 2.3. Data Collection Tools and Methods

Data were collected through structured, interviewer-administered questionnaires conducted by trained fieldworkers. To ensure linguistic accessibility, the English-language instrument included key terminology pre-translated into isiZulu and Sesotho, the two common local languages. A multilingual epidemiologist conducted the translations; however, formal back-translation or piloting was not conducted. This is acknowledged as a limitation that may affect the linguistic validity of the instrument. Fieldworkers received standardised training on questionnaire administration, including translation of difficult terms and concepts, prior to data collection. After written informed consent was obtained, fieldworkers captured participant responses in real time using password-protected tablets. This process utilised the REDCap (Research Electronic Data Capture) platform, a secure web-based application designed for encrypted data management and validated research collection [13].

The binary outcome variable, self-reported workplace injury in the last 6 months, was made up of the question: “In the last 6 months, have you experienced (a) cuts by material, (b) needle-stick injury, (c) attacks by dogs, (d) bites by rodents, (e) injury by waste fall, (f) injury by vehicle, or (g) violence by another waste picker”. Participants who reported at least one of these injuries were categorised as “yes”; otherwise, “no”.

Consistent with previous literature [7], eleven exploratory variables were selected and categorised for analysis. Demographic and socioeconomic factors included gender (male or female), age, highest educational attainment (no schooling, primary, secondary, or tertiary), and average monthly income. Occupational and behavioural variables comprised current or ever smoked and alcohol use (dichotomised as yes/no), average daily working hours, and total years of experience at landfill sites. Health-related factors were identified through self-reported history of chronic diseases (defined as having either diabetes, hypertension, asthma, cancer, or stroke) and the presence of sensory impairments (hearing or vision problems). Lastly, the use of Personal Protective Equipment (PPE) was defined as the use of at least one protective item, such as a mask, gloves, or safety footwear.

### 2.4. Statistical Analysis

Statistical analysis was performed using Stata version 19.5 (StataCorp, College Station, TX, USA). Baseline characteristics, including socio-demographic and occupational profiles, were analysed using descriptive statistics; categorical data were presented as frequencies and percentages, while continuous variables were summarised as medians and quartiles (25th and 75th percentiles). Missing data were handled using complete case analysis. Of the 354 descriptive-analytic sample, the final multivariable model retained 350 observations, with the four-participant reduction reflecting covariate-level missingness. To investigate factors associated with the prevalence of self-reported workplace injuries over the preceding six months, robust (modified). Poisson regression models were utilised. This approach was selected over binary logistic regression because odds ratios tend to overestimate effect sizes when the outcome of interest is common (prevalence > 10%) [14]. A forward stepwise approach was used for variable selection, with a screening threshold of *p* < 0.25 applied in univariable analyses. This threshold was adopted as a liberal screening criterion to retain variables that may act as confounders even in the absence of a strong marginal association with the outcome [15]. In the regression models, measures of association were reported as crude and adjusted prevalence ratios (aPRs) with corresponding 95% confidence intervals (95% CI). A post hoc sensitivity analysis was additionally conducted with the landfill site forced into the multivariable model to assess its potential confounding influence. All tests were two-sided, and a *p*-value of <0.05 was considered the threshold for statistical significance in the final analysis. Model fit was assessed using the Pearson chi-square goodness-of-fit statistic and deviance.

### 2.5. Ethical Considerations

Ethical approval for this study was granted by the Human Research Ethics Committee (HREC) of the Faculty of Health Sciences at the University of the Witwatersrand (Certificate No. M171120). Administrative permission to access the study sites was obtained from the respective landfill management authorities prior to data collection. Prior to consent, participants were explicitly informed that participation was entirely voluntary, that refusal or withdrawal would in no way affect their access to or status at the landfill site, and that their responses would not be shared with landfill management authorities. Interviews were conducted away from site management personnel to minimise any perception of institutional oversight. A token of appreciation of R50 was offered for participation as per ethics requirements. To protect participant anonymity, no personally identifiable information was recorded during the interview process. Confidentiality was maintained by assigning each participant a unique identifier.

## 3. Results

Table 1 presents the socio-demographic characteristics, occupational history, and health status of the study participants across the two landfill sites. The majority of waste pickers in this study were male (n = 259; 73.2%), although gender distribution varied substantially by site. Landfill Site 1 was predominantly male (n = 229; 78.4%), while Landfill Site 2 had a nearly equal gender split (n = 32; 51.6% female). The largest age group was the 29–39 year category (n = 152; 42.9%), with noticeable variation between the two sites. Educational attainment was high, with 77.6% (n = 274) having completed secondary school, while only 1.4% (n = 5) had reached tertiary education. The participants had worked at the landfill sites for a median of 5 years (Q1, Q3: 3.0, 10.0), though workers at Site 2 had much longer median experience of 12.5 years. The median total working time was 7 h per day (Q1, Q3: 5.0, 8.0), but workers at Site 1 reported longer daily shifts (median 7 h) than those at Site 2 (median 4 h). The median monthly income for the total sample was ZAR 1500 (Q1, Q3: 850.0, 2200.0) [91.56 USD; 52.23, 135.98]. Regarding lifestyle characteristics, more than two-thirds of the participants reported ‘yes’ to current or ever smoked (n = 246; 69.5%), and 42.1% (n = 115) reported alcohol use. Over a tenth (n = 48; 13.6%) of the study participants had a chronic disease, and sensory impairments, specifically hearing or vision problems, were prevalent in 22.0% (n = 78) of the participants. Lastly, 86.2% (n = 305) of the waste pickers had at least one self-reported workplace injury in the prior six months.

Table 2 summarises the prevalence of PPE use and the distribution of various occupational injuries reported over the preceding six months. The majority of landfill waste pickers reported utilising at least one form of PPE (94.1%; n = 333). Lacerations caused by contact with waste materials were the most pervasive, affecting 82.7% (n = 291) of the waste pickers. This was followed by one in five participants (20.5%; n = 71) reporting experiencing injuries due to violence involving other waste pickers. One-fifth of participants (19.9%; n = 70) reported needle-stick injuries, and 19.4% (n = 68) had been injured by waste falls. Additionally, 10.0% (n = 35) of workers reported dog attacks, vehicle-related incidents accounted for nearly 9.4% of reported injuries (n = 33), and 5.8% (n = 20) reported rodent bites. The listed injury categories are heterogeneous and reflect distinct mechanisms of harm.

Table 3 presents the univariable and multivariable prevalence ratios (PR) for factors associated with self-reported workplace injuries. In the univariable analyses, belonging to the oldest age group (51+ years) was significantly associated with a lower injury prevalence than the 18–28 age group (PR 0.77; 95% CI: 0.59–0.99). While monthly income reached statistical significance, the effect size was negligible (PR 1.00; 95% CI: 1.00–1.00). In the final multivariable model, 350 observations were retained, and model fit indicated no evidence of poor fit (*p* = 1.000). In the adjusted model, waste pickers aged 51 and older had a 32% lower prevalence of reporting an injury than those in the 18–28 age group (aPR 0.68; 95% CI: 0.51–0.90). In addition, for each additional year of experience, the prevalence of reporting injuries increased by approximately 1.0% (aPR 1.01; 95% CI: 1.01–1.02). Gender, intermediate age categories, and other occupational factors did not reach statistical significance in the adjusted model. As a sensitivity analysis, the landfill site was forced into the multivariable model alongside the retained covariates, despite not meeting the *p* < 0.25 screening threshold in univariable analysis (PR 0.99; 95% CI 0.86–1.10). The site coefficient was small and non-significant (aPR 0.94; 95% CI 0.82–1.06; *p* = 0.307), and the primary findings were robust to this adjustment: the protective association with age 51 years and older (aPR 0.681; 95% CI 0.512–0.905) and the positive association with years of landfill experience (aPR 1.016; 95% CI 1.006–1.025) were materially unchanged. These results indicate that the site did not act as a meaningful confounder in the adjusted model.

## 4. Discussion

This study describes the prevalence and associated risk factors of non-fatal workplace injuries among informal waste pickers at landfill sites in Johannesburg, South Africa. Our findings highlight the physical risks inherent in the informal recovery sector, particularly within the unregulated environment of municipal dumps. We found a high self-reported injury prevalence of 86.2%, with lacerations alone affecting 82.7% of the participants in the previous six months. The injury prevalence in this study (86.2%) exceeded the 69.4% reported among waste recyclers at Buy-Back Centres (BBCs) in Johannesburg [7]. This disparity likely reflects the difference between the semi-formalised, intermediate environment of BBCs, which often offer designated sorting areas and PPE, and the hazardous, unpredictable nature of landfill sites where pickers operate directly amidst heavy machinery and raw waste. However, this comparison should be interpreted cautiously, as differences may reflect not only environmental settings but also differences in study populations, injury definitions, work organisation, and the period of data collection. Notably, the broader injury definition used in the present study, which includes violence and animal attacks, may contribute to the higher observed prevalence. Our findings are consistent with studies of landfill waste pickers in other developing contexts, such as the 78.6% prevalence reported in Nigeria [8].

The most common injuries reported in our study were cuts from materials (82.7%), followed by violence from other waste pickers (20.5%), and needle-stick injuries (19.9%). Although of a different magnitude, cuts from materials such as metal, broken glass and razor blades have been reported as the most frequent injuries among landfill waste pickers in Nigeria (37.8%) [8]. The high frequency of lacerations and needle-stick injuries is consistent with repeated manual contact with mixed waste and sharp materials in landfill settings. These findings may reflect inadequate waste segregation at source and unsafe disposal of sharps, the latter being consistent with evidence that improper sharps disposal is prevalent in South African communities [16], although the present study did not directly assess these upstream waste-management practices. The reported levels of interpersonal violence (20.5%) and animal attacks (10.0%) further suggest that occupational harm in this setting extends beyond mechanical injury alone. The prevalence of violence-related injuries (20.5%) warrants careful interpretation. In a context where competition for high-value recyclables is intense and unregulated, minor physical conflicts may be normalised and therefore underreported, suggesting that the true prevalence may be higher than recorded. The drivers of interpersonal conflict in landfill settings are likely multifactorial: competition for early access to incoming waste trucks, territorial arrangements over productive sorting areas, and disputes over recovered materials are all plausible sources of conflict. These dynamics are consistent with the broader literature on resource competition in informal economies, though the specific contribution of these dynamics to injury risk was not directly assessed in this study. Future research incorporating qualitative methods would be well-positioned to explore the social and organisational drivers of conflict-related injury in this setting.

Despite 94.1% of participants reporting use of at least one form of PPE, injury prevalence remained high. Our study measured whether PPE was used, not its type, condition, frequency, or appropriateness to the task. It is therefore not possible to draw conclusions about PPE adequacy from this data. The observation nonetheless highlights the need for future research to assess PPE quality and suitability beyond binary indicators of use. Similar observations have been made in other Southern African contexts, where despite high PPE awareness, workers often use inadequate gear due to resource constraints or discomfort [7]. In our adjusted multivariable model, we observed that for every one-year increase in work experience at the landfill, the prevalence of reporting injury increased by 1.0%. This finding is in line with a study from Ethiopia that showed that solid waste collectors with more than 1 year of work experience have higher odds of occupational injury (aOR = 5.98; 95% CI: 2.01–17.75) [17]. In contrast, pickers aged 51 and older were 32% less likely to report an injury than those aged 18–28, mirroring global trends in which younger, less experienced workers sustain more injuries due to high-intensity work patterns and a lack of safety training [18]. Nonetheless, this statistic was inconsistent with studies conducted in the Republic of Korea and South Africa, which showed that most injuries occurred in older workers—albeit among household waste collectors and BBC waste recyclers, respectively [7,19].

The simultaneous presence of a positive experience–injury association and a protective age effect is not inherently contradictory, as these variables operate independently in the multivariable model. Workers with many years of landfill experience are not necessarily older; many may have entered this work in early adulthood, and the model estimates each effect while adjusting for the others. The modest incremental association between experience and injury prevalence (aPR 1.01 per year) may reflect cumulative exposure to hazardous conditions, while the protective effect observed in the oldest age group may reflect factors not captured in this analysis. One plausible explanation is that older workers may occupy more established positions within informal workplace hierarchies, potentially affording them greater control over task allocation and reducing their exposure to the most hazardous activities. However, none of these mechanisms was directly measured and should be understood as hypotheses to be examined in future qualitative and longitudinal work. Taken together, these findings have several implications for occupational health policy and practice, though the cross-sectional design limits causal inference and the following recommendations should be understood as evidence-informed priorities rather than established conclusions.

Municipal integration of informal landfill pickers could include formal registration of workers at each site to enable surveillance and accountability; mandatory induction, health and safety training as a condition of site access; provision of task-appropriate PPE through site management rather than reliance on workers sourcing their own equipment; and inclusion of waste pickers in occupational health monitoring programmes. Staffing implications for municipalities include designating waste management, health, and safety officers to oversee informal workers and establishing referral pathways to occupational health services. These measures would require dedicated budget lines within municipal waste management frameworks, which are currently absent in most South African municipalities. Training should be task-specific and address the primary hazard types identified in this study: safe handling of sharp and mixed waste materials; recognition and avoidance of sharps; procedures during vehicle manoeuvring; and de-escalation of interpersonal conflict. Refresher training should be provided periodically rather than offered solely at induction.

To our knowledge, this is one of the few assessments of the informal landfill workers in Johannesburg. The evidence is clear: the informal landfill sector is a high-risk environment that requires urgent integration into municipal health and safety frameworks. Despite the significance of these findings, this study has limitations. The cross-sectional design of the study prevents establishing a causal relationship between specific work habits and injury. Furthermore, the use of a convenience sampling method may limit the generalisability of the findings to the broader population of informal waste pickers in other regions. The reliance on self-reported data introduces potential “recall bias” or “social desirability bias,” where participants may underreport injuries due to stigma or fear of losing access to the site. Additionally, our definition of injury was deliberately broad and did not account for the clinical severity of the incidents; consequently, the inclusion of minor cuts or lacerations likely contributed to the high prevalence rates observed in this group. Lastly, the study did not capture data on the anatomical location of injuries. Body mapping would have provided clinically and ergonomically meaningful data to complement the injury-type prevalences reported here, and should be incorporated into future studies of this population.

Future research should utilise longitudinal designs to track injury over time and account for seasonal variations in waste types. Furthermore, incorporating direct workplace observations would help validate the quality, fitness, and actual usage of PPE beyond self-reported metrics. Researchers should also conduct qualitative inquiries into the “economic-safety trade-off” to understand if pickers discard PPE when it hinders their sorting speed and daily income. Although this study was restricted to participants aged 18 years and older, it is important to acknowledge that children may be present in landfill communities where residential and occupational environments overlap. Informal waste picking is not exclusively an adult activity, and children residing on or near landfill sites may be exposed to the same mechanical, biological, and social hazards documented in this study, or may participate in waste-picking activities alongside adult family members. This represents a distinct and significant vulnerability dimension that was beyond the scope of the current study but warrants dedicated attention in future research, child protection policy, and municipal waste management frameworks. Finally, trialling tailored, industrial-grade puncture-resistant interventions in controlled settings remains a priority for evidence-based policymaking.

## 5. Conclusions

This study found a high prevalence of self-reported non-fatal workplace injury among informal waste pickers working at landfill sites in Johannesburg, with cuts, needle-stick injuries, and violence-related injuries being among the most frequently reported events. Our findings suggest that each additional year of landfill work experience was associated with a modest increase in the prevalence of reported injury. Whether this reflects increased total exposure, changes in work behaviour, or altered hazard perception was not directly measured. Nonetheless, we recommend implementing health and safety training for all informal waste pickers as a prerequisite for site entry. This should be coupled with the provision of required, task-appropriate, puncture-resistant PPE and with consideration for the formal integration of these workers into municipal waste management frameworks. Such integration would allow for better site regulation, reduced interpersonal conflict, and the recognition of informal pickers as essential environmental workers who deserve comprehensive, institutionalised occupational health protections.

## Figures and Tables

**Table 1:** Description of socio-demographic, occupational, lifestyle, and health outcomes by landfill site.

	Landfill Site 1	Landfill Site 2	Total
	(N = 292)	(N = 62)	(N = 354)
**Gender**			
Male	229 (78.4%)	30 (48.4%)	259 (73.2%)
Female	63 (21.6%)	32 (51.6%)	95 (26.8%)
**Age category**			
18–28 Years	118 (40.4%)	5 (8.1%)	123 (34.7%)
29–39 Years	129 (44.2%)	23 (37.1%)	152 (42.9%)
40–50 Years	29 (9.9%)	18 (29.0%)	47 (13.3%)
51+ Years	16 (5.5%)	16 (25.8%)	32 (9.0%)
**Level of education obtained**			
No schooling	11 (3.8%)	4 (6.5%)	15 (4.2%)
Primary school	35 (12.0%)	24 (38.7%)	59 (16.7%)
Secondary school	241 (82.8%)	33 (53.2%)	274 (77.6%)
Tertiary level	4 (1.4%)	1 (1.6%)	5 (1.4%)
**Monthly income in rands**			
Median (Q1, Q3)	1400.0 (800.0, 2000.0)	2000.0 (1000.0, 2500.0)	1500.0 (850.0, 2200.0)
**Hours worked per day**			
Median (Q1, Q3)	7.0 (6.0, 8.0)	4.0 (2.0, 6.8)	7.0 (5.0, 8.0)
**Years working in a landfill site**			
Median (Q1, Q3)	4.0 (2.0, 7.0)	12.5 (9.0, 17.0)	5.0 (3.0, 10.0)
**Current or ever smoked**			
No	72 (24.7%)	36 (58.1%)	108 (30.5%)
Yes	220 (75.3%)	26 (41.9%)	246 (69.5%)
**Alcohol use**			
No	142 (60.7%)	16 (41.0%)	158 (57.9%)
Yes	92 (39.3%)	23 (59.0%)	115 (42.1%)
**Chronic diseases**			
No	253 (86.6%)	53 (85.5%)	306 (86.4%)
Yes	39 (13.4%)	9 (14.5%)	48 (13.6%)
**Have hearing and or vision problems**			
No	229 (78.4%)	47 (75.8%)	276 (78.0%)
Yes	63 (21.6%)	15 (24.2%)	78 (22.0%)
**Injured at work in the last 6 months**			
No	39 (13.4%)	10 (16.1%)	49 (13.8%)
Yes	253 (86.6%)	52 (83.9%)	305 (86.2%)

Variables education and alcohol use do not add up to the total (N = 354) due to missing responses.

**Table 2:** Description of self-reported PPE use and workplace injuries in the last 6 months.

	Total (N = 354)
**Use of PPE**	
No	21 (5.9%)
Yes	333 (94.1%)
**Cut by materials**	
No	61 (17.3%)
Yes	291 (82.7%)
**Needle stick injuries**	
No	282 (80.1%)
Yes	70 (19.9%)
**Attacked by dogs**	
No	316 (90.0%)
Yes	35 (10.0%)
**Bitten by rodents**	
No	327 (94.2%)
Yes	20 (5.8%)
**Injured by a waste fall**	
No	282 (80.6%)
Yes	68 (19.4%)
**Injured by a vehicle**	
No	319 (90.6%)
Yes	33 (9.4%)
**Violence by other waste pickers**	
No	276 (79.5%)
Yes	71 (20.5%)

All variables, except for the use of PPE, do not add up to the total (N = 354) due to missing responses.

**Table 3:** Factors associated with self-reported workplace injuries.

Variable	Univariable Analyses PR (95% CI)	Multivariable Analyses aPR (95% CI)
**Landfill site**		
Site 1	1 (ref)	
Site 2	0.99 (0.86; 1.10)	
**Gender**		
Male	1 (ref)	1 (ref)
Female	0.99 (0.89; 1.09)	1.02 (0.92; 1.13)
**Age group**		
18–28 years	1 (ref)	1 (ref)
29–39 years	1.07 (0.98; 1.17)	1.04 (0.95; 1.14)
40–50 years	1.00 (0.87; 1.14)	0.93 (0.79; 1.08)
51+ years	0.77 (0.59; 0.99) *	0.68 (0.51; 0.90) *
**Level of education**		
No schooling	1 (ref)	
Primary school	1.16 (0.84; 1.60)	
Secondary school	1.19 (0.87; 1.62)	
Tertiary school	1.09 (0.64; 1.86)	
**Monthly income**	1.00 (1.00; 1.00) *	
**Hours worked per day**	1.00 (0.98; 1.01)	
**Years working in a landfill site**	1.00 (1.00; 1.01)	1.01 (1.01; 1.02) *
**Current or ever smoked**		
No	1 (ref)	
Yes	1.07 (0.97; 1.18)	
**Alcohol use**		
No	1 (ref)	
Yes	0.97 (0.88; 1.07)	
**Chronic disease/s**		
No	1 (ref)	
Yes	1.02 (0.91; 1.14)	
**Hearing and or vision problems**		
No	1 (ref)	
Yes	1.03 (0.94; 1.14)	
**Use of PPE**		
No	1 (ref)	
Yes	1.07 (0.86; 1.32)	

* *p* < 0.05; CI: Confidence intervals; PR: Prevalence ratio; aPR: Adjusted prevalence ratio; PPE: Personal protective equipment; ref: reference.

## Data Availability

The data supporting this study’s findings are available upon request from N.N.
